# Emerging role of the TCA cycle and its metabolites in lung disease

**DOI:** 10.3389/fphys.2025.1621013

**Published:** 2025-08-15

**Authors:** Xinan Lu, Aijun Zhang, Huaying Wang, Xinjia Xu, Liping Chen, Lingfang Luo

**Affiliations:** ^1^ Department of Respiratory and Critical Care Medicine, Affiliated Cixi Hospital, Wenzhou Medical University, Ningbo, Zhejiang, China; ^2^ Department of Respiratory and Critical Care Medicine, Affiliated People’s Hospital, Ningbo University, Ningbo, Zhejiang, China

**Keywords:** TCA cycle, asthma, chronic obstructive pulmonary disease (COPD), lung infection, pulmonary hypertension (PH), lung cancer

## Abstract

The TCA cycle is a central pathway for oxidative phosphorylation in eukaryotic mitochondria, plays a role in energy production and biosynthesis, and can alter cellular function in a variety of ways. As our understanding of the TCA cycle has improved, there is increasing evidence that it is inextricably linked to lung disease. This article summarizes the relationship between the TCA cycle and different types of lung disease and focuses on the specific mechanisms by which the TCA cycle and its intermediate metabolites influence lung disease progression.

## 1 Introduction

Mitochondria play a pivotal role in eukaryotes, facilitating cellular energy production and biosynthesis; they regulate the energy metabolism essential for cell growth, primarily through the tricarboxylic acid cycle (TCA cycle) and oxidative phosphorylation (Koranteng et al., 2024). In the cytoplasm, glucose is converted to pyruvate through glycolysis. In the presence of sufficient oxygen, pyruvate enters the mitochondria and is completely oxidized to carbon dioxide and water through the TCA cycle, generating high-energy electrons and intermediary metabolites of the TCA cycle (Wu et al., 2024).

The TCA cycle and its metabolic intermediates are involved in a variety of key biological processes and can influence biological pathophysiology by regulating NADH/NADPH homeostasis, scavenging reactive oxygen species (ROS), and generating ATP through phosphorylation at the substrate level (MacLean et al., 2023). Further studies have indicated that the TCA cycle may play a role in the progression of lung diseases. This article provides a review of the role of the TCA cycle in the development of lung diseases and the related mechanisms, with the goal of providing new insights into the diagnosis and treatment of lung diseases.

### 1.1 The TCA cycle and its intermediate metabolites

The TCA cycle, also referred to as the citric acid cycle, commences with the incorporation of acetyl coenzyme A (acetyl-CoA) into the cycle via glycolysis, fatty acid beta-oxidation, or amino acid metabolism; this represents the principal mechanism through which mitochondrial energy is produced (Wu et al., 2024). Acetyl-CoA enters the circulation and combines with oxaloacetate to form citric acid in the presence of citrate synthase. The conversion of citric acid to its isomer isocitrate is subsequently catalyzed by cis-aconitase. The cycle continues with two oxidative decarboxylation reactions in which isocitrate is converted into five-carbon α-ketoglutarate (α-KG) and subsequently into four-carbon succinyl coenzyme A with the release of two molecules of CO_2_ and the generation of two NADH molecules. Subsequently, succinyl-CoA hydrolyses the thioester bond to produce succinate by thiokinase, releasing GTP to produce ATP. In the subsequent phase, succinate is oxidized to fumarate by succinate dehydrogenase (SDH). Succinate oxidation leads to FADH2 reduction in SDHA, which transfers electrons to iron sulfur clusters in SDHB, this electrons will then travel across electron transport chain (ETC.). Following the conversion of fumarate to malic acid, the latter subsequently undergoes further dehydrogenation, resulting in the production of NADPH and oxaloacetic acid, which then enter the tricarboxylic acid cycle once more. The TCA cycle is a metabolic pathway that not only generates energy and electron carriers (such as NADH2 AND FADH2) but also produces a variety of intermediate metabolites. These metabolites play a role in controlling the chromatin modification of proteins, DNA methylation, and posttranslational modification, which can alter cellular function (Martínez-Reyes and Chandel, 2020). The TCA cycle metabolites that influence cellular function include citrate, α-KG, succinate, fumarate, and itaconate.

Citrate plays a role in protein acetylation and fatty acid synthesis on the one hand and in regulating energy metabolism on the other hand (Icard et al., 2023). Mutations in SDH result in the accumulation of succinate, a compound commonly found in a variety of cancers. Consequently, succinate is regarded as a cancer metabolite (Dalla Pozza et al., 2020). Furthermore, succinate plays a pivotal role in the regulation of innate immunity. Metabolic analyses of LPS-treated macrophages have revealed a considerable abundance of succinate within these cells (Mills et al., 2016). α-KG is generated from isocitrate via the action of isocitrate dehydrogenase and can serve as substrate for glutamine synthesis, forming a crucial link between the TCA cycle and glutamine metabolism (Park et al., 2023). Furthermore, α-KG serves as a pivotal regulator of the hypoxic response (Tennant et al., 2009) and plays a significant role in the inflammatory response (Liu et al., 2017). Fumarates promote tumor growth through multiple signal transduction pathways and act as immunomodulators, exerting their effects by controlling chromatin modifications (Martínez-Reyes and Chandel, 2020). Itaconate is derived from the decarboxylation of cis-aconitic acid and serves as an important immunomodulator and antimicrobial agent (Bambouskova et al., 2018). Itaconate activates the downstream pathway of the antioxidant transcription factor NRF2, thereby contributing to its anti-inflammatory properties in activated macrophages (Mills et al., 2018).

In lung disease, The TCA cycle metabolites that influence lung disease include citrate, α-KG, succinate, fumarate, and itaconate. The present paper sets out the findings that elevated levels of succinate, fumarate and malate have been detected in patients suffering from chronic obstructive pulmonary disease (COPD), tuberculosis (TB) and lung cancer (LC) (Pinto-Plata et al., 2019; Collins et al., 2021; Alosaimi et al., 2024) It has been observed that levels of fumarate and malate decrease in cases of pulmonary hypertension, while citrate and succinate levels increase (Sakao et al., 2021; Zhao et al., 2014). The detailed exposition of the specific impact mechanisms under consideration will be provided in the following section.

### 1.2 The TCA cycle and chronic inflammatory airway disease

Chronic inflammatory airway disease is defined as a chronic condition in which there is inflammation of the upper and/or lower airways. The hallmark symptoms of this disease are airway inflammation, obstruction and remodeling. The most common forms of chronic inflammatory airway disease are asthma and COPD (Mannion et al., 2023).

#### 1.2.1 The influence between the TCA cycle and bronchial asthma

**FIGURE 1:**
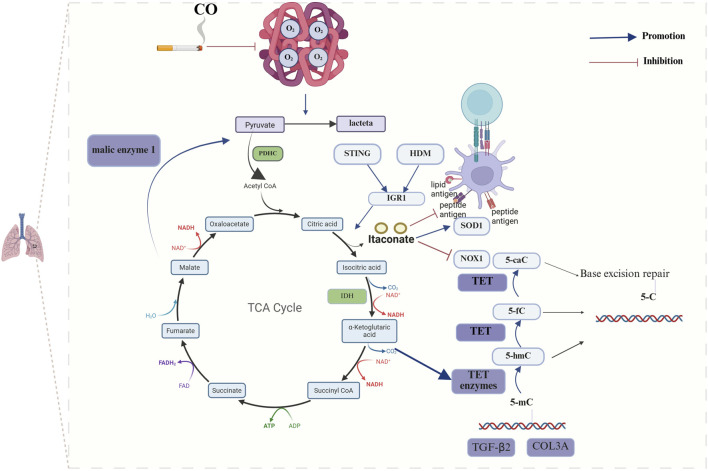
Mechanisms of TCA cycle metabolites in chronic airway inflammatory diseases. The development of bronchial asthma significantly influences disease progression, primarily through its impact on the abundance of itaconic acid and α-KG within the TCA cycle. Elevated expression of IDH results in the increased expression of α-KG, which in turn facilitates the activation of TET enzymes and ultimately induces the hypomethylation of specific genes, including TGF-β2 and COL3A. HDMs or STING pathway stimulation catalyzes the production of the itaconate by inducing the expression of IRG1. The IRG1/itaconate pathway decreases the allergen uptake and antigen presentation capacity of DCs to CD4^+^ T cells and influences the expression of SOD1 and NOX1. The progression of COPD is significantly influenced by the interplay between pyruvate and oxidative stress. CO reduces the oxygen-binding capacity of hemoglobin, which leads to a reduction in the overall activity of aerobic pathways and an increase in anaerobic glycolysis activity. (The blue arrows represent promotion, and the red arrows represent inhibition; created using BioRender.com).

Bronchial asthma is a chronic respiratory disease that is characterized by four main features: airway inflammation, bronchial hyperresponsiveness, increased mucus production and varying degrees of airflow limitation (Barosova et al., 2023). On the basis of the presence or absence of eosinophil recruitment, bronchial asthma can be classified into two main categories: a type-2 (T2 or Th2)-high endotype with a type 2 immune response and eosinophil recruitment and a type-2 (T2 or Th2) endotype with activation-mediated activation of Th1 and/or Th17 cells, which is considered a low or nontype-2 endotype (Kuruvilla et al., 2019). Research has indicated a potential correlation between bronchial asthma and the TCA cycle. Metabolomic analyses of ovalbumin (OVA)-induced asthmatic mice have demonstrated that organic acid metabolism is altered in these mice compared with that observed in control mice (Seo et al., 2017). Furthermore, metabolomic analysis of urine from children with asthma revealed a potential correlation between the TCA cycle and the pathogenesis of asthma (Li S. et al., 2020). Further mechanistic studies have demonstrated that elevated expression of isocitrate dehydrogenase (IDH) results in the increased expression of α-KG within the TCA cycle, which in turn facilitates the activation of ten-eleven translocation (TET) enzymes. The activation of TET enzymes has been shown to induce the hypomethylation of specific genes, including transforming growth factor-β2 (TGF-β2) and type III collagen (COL3A), which are characteristic of smooth muscle cells in the airways of individuals with asthma (Huang, 2020). Furthermore, it has been demonstrated that house dust mites (HDMs) alone or STING pathway stimulation can progressively activate the expression of immune responsive gene 1 (IRG1) in dendritic cells via the mitochondrial superoxide-dependent pathway. Additionally, IRG1 has been shown to catalyze the production of the TCA cycle intermediate itaconate. The IRG1/itaconate pathway, on the one hand, attenuates the allergen uptake and antigen presentation capacity of DCs to CD4^+^ T cells; on the other hand, it can promote the expression of superoxide dismutase 1 (superoxide radical scavenger) and inhibit the expression of NADPH oxidase 1, a superoxide-generating oxidase, through redox metabolism, thus reducing the production of mitochondrial superoxide. In a previous study, exogenous itaconate (4-octyl itaconate) was administered via the intrapulmonary route, and 4-OI attenuated the features of allergic asthma manifestations, including HDM sensitization, Th2-mediated eosinophilic airway inflammation, and mucosal cellular hyperplasia (Jaiswal et al., 2022) (Figure 1).

#### 1.2.2 The influence between the TCA cycle and COPD

COPD is characterized by chronic bronchitis, mucosal ciliary dysfunction, emphysema and a progressive decline in lung function. Tobacco smoke exposure represents the primary environmental risk factor for the development of COPD. However, bacterial and viral infections frequently lead to acute exacerbations (Mammen and Sethi, 2016; Schleich et al., 2023). Research has demonstrated that the activation of the TCA cycle results in increased ATP production and that elevated extracellular ATP levels within the airway lumen are linked to the pathogenesis of COPD (Naz et al., 2017). Furthermore, the TCA cycle intermediates have been shown to influence the survival of COPD patients. Plasma metabolomics sequencing data from COPD patients suggests that α-KG, succinate, fumarate, and malate are significantly higher in patients who succumb to COPD than in those who survive the disease (Pinto-Plata et al., 2019). To gain deeper insight into the interrelationship between the TCA cycle and COPD, researchers have conducted further mechanistic studies and discovered, through RNA sequencing, that the expression of malic enzyme 1 (ME1) transcripts is downregulated in alveolar macrophages (AMs) of COPD patients compared with healthy donors. ME1 catalyzes the reversible oxidative decarboxylation of malate to pyruvate, thereby replenishing TCA cycle intermediates and converting NADP + to NADPH. Conversely, ME1 deletion results in a lower oxygen consumption rate (OCR)/extracellular acidification rate (ECAR) ratio and a reduction in TCA cycle intermediates (Ryan et al., 2023). Smoking represents a significant environmental factor in the development of COPD. Carbon monoxide in smoke significantly reduces the oxygen-binding capacity of hemoglobin. Additionally, lung parenchymal injury due to oxidative stress and a chronic inflammatory response cause respiratory and locomotor muscles to atrophy to varying degrees, which reduces the capacity for oxygen uptake. Compared with that in healthy controls, the combination of these two factors results in a hypoxic state in individuals with COPD who smoke; this leads to a reduction in the overall level of aerobic pathways and an increase in anaerobic glycolysis, which in turn results in dysregulation of the TCA cycle. The degree of this dysregulation is correlated with the severity of the disease (Xue et al., 2021) (Figure 1).

The literature indicates that the TCA cycle plays a significant role in the progression and treatment of chronic airway inflammatory diseases, including bronchial asthma and COPD. However, few studies have explored the mechanisms through which the TCA cycle affects chronic airway inflammatory diseases. Therefore, further studies focusing on the mechanism of the TCA cycle in chronic airway inflammatory diseases are needed to provide new insights and ideas for the treatment of such diseases.

### 1.3 The TCA cycle and infectious diseases of the lungs

Lung infections represent a significant global health burden, with considerable impacts on both lung health and the exacerbation of other chronic inflammatory airway diseases, including asthma and COPD. Additionally, they can lead to the development of other systemic diseases, such as cardiovascular diseases. Both bacterial and viral result in a wide range of lung infections, varying in severity (Hambo and Harb, 2023; Fitzpatrick and Busse, 2022). Lung infections progress through distinct stages. Upon the establishment of an infectious focus, bacteria undergo metabolic alterations and virulence regulation to increase their ability to survive in the nutrient-limited and oxidative environment of the airways (Tomlinson et al., 2021).

#### 1.3.1 The influence between the TCA cycle and bacterial infection

**FIGURE 2:**
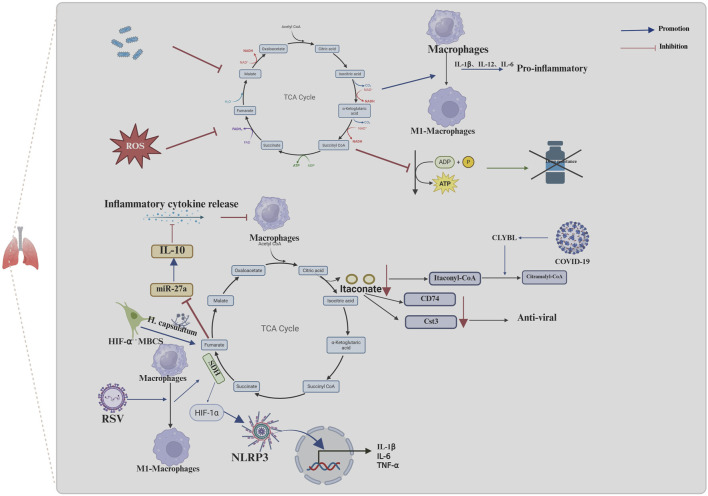
Mechanisms of the TCA cycle and its metabolites in infectious diseases of the lungs. Macrophage effects on the TCA cycle in infectious lung disease promote infection progression and drug resistance. The inhibition of the TCA cycle by TB and ROS, promotes the release of inflammatory factors through promoting the conversion of macrophages to pro-inflammatory macrophages (M1-macrophages) and inhibits the production of ATP, which can cause resistance to drugs. COVID-19 leads to greater conversion of itaconyl-CoA to citramalyl-CoA mediated by upregulation of CLYBL, which in turn, promotes the conversion of itaconate to itaconyl-CoA and results in a decrease in itaconate. The decrease of itaconate decreases the expression of CD74 and Cst3, in macrophages; this, in turn, suppresses the antiviral effect. RSV promotes the conversion of macrophages to M1-macrophages and then stimulates the activity of SDH in the TCA cycle, which in turn facilitates the release of cytokines, such as IL-1β, through HIF-1α/NLRP3 pathway and contributes to the host defense response, stimulating lung tissue inflammation or injury. In the absence of HIF-1α, lung infection by *Histoplasma capsulatum* in MBCS resulted in the accumulation of fumarate, which further promoted IL-10 protein production by downregulating miR-27a expression IL-10 accumulation inhibits the ability of macrophages to respond to proinflammatory cytokines. (The blue arrows represent promotion, and the red arrows represent inhibition; created using BioRender.com).

The TCA cycle is a crucial metabolic pathway in mitochondria. Previous studies have investigated the metabolomic profile of urine from mice infected with *Streptococcus pneumoniae*, a bacterium that commonly causes lung infections. These studies revealed a notable reduction in TCA cycle intermediates in the urine of mice treated with *S. pneumoniae* (Slupsky et al., 2009). Additionally, TCA cycle remodeling was identified in patients with both active and untreated TB; this was characterized by elevated plasma concentrations of the TCA cycle intermediates succinate, fumarate and malate and reduced concentrations of itaconate. Furthermore, this effect was more pronounced in patients with multidrug-resistant TB. However, the administration of appropriate antituberculosis treatment was observed to reverse this proinflammatory response to a certain extent. Further mechanistic studies have demonstrated that TCA cycle remodeling is a key driver of inflammation, whereby the upregulation of IL-1β expression and downregulation of granulocyte–macrophage colony-stimulating factor (GM-CSF) expression facilitate the production of proinflammatory arachidonic acid (Collins et al., 2021). Furthermore, in a mouse model of sepsis, the polarization of pulmonary M1-type macrophages is accompanied by a reduction in PRDX3 expression (Huang et al., 2024). The present study investigates the function of PRDX3 in relation to mitochondrial hydrogen peroxide metabolism, with the aim of elucidating its role in oxidative stress and cellular damage (Cox et al., 2009; Cadenas, 2004). Further investigation into the effects of PRDX3 revealed an increase in circulating metabolites in TCA and a shift in M1-type macrophages towards a M2-type macrophages. (Huang et al., 2024). In addition to its impact on the progression of lung infections, the TCA cycle influences the efficacy of subsequent antibiotic therapy in the context of bacterial infections. ROS produced by macrophages can inactivate the TCA cycle by inactivating essential TCA cycle enzymes, such as aconitase and succinate dehydrogenase. This leads to a decrease in respiration and ATP production, which ultimately increases the antibiotic resistance of *Staphylococcus aureus* (Rowe et al., 2020) (Figure 2).

#### 1.3.2 The influence between the TCA cycle and viral infection

Viruses represent a significant additional cause of lung infections. Severe viral infections can result in excessive inflammation, lung damage, acute respiratory distress syndrome and, in the most severe cases, death (Gautam et al., 2024). Coronavirus disease 2019 (COVID-19) has become one of the most prevalent viruses in recent years. Metabolomic analyses of plasma samples from patients with prolonged symptoms following COVID-19 infection revealed alterations primarily associated with increased TCA cycle activity (Martínez et al.). Further mechanistic studies revealed that in patients with severe manifestations of COVID-19 infection, the specific upregulation of Citramalyl-CoA lyase (CLYBL) leads to greater conversion of itaconyl-CoA to citramalyl-CoA by promoting the cleavage of citramalyl-CoA. This, in turn, promotes the conversion of itaconate to itaconyl-CoA, which results in a decrease in itaconate (Liu et al., 2022). In contrast, itaconate has been shown to upregulate the expression of proteins with antiviral and immune functions, including CD74 and cystatin 3 (Cst3), in macrophages; this, in turn, has been shown to exert an inhibitory effect on viral entry into cells, regulate immunity and provide cytoprotection (Bruchez et al., 2020; Jakoš et al., 2019; Li R. et al., 2020). In addition to the effects of the COVID-19 virus, respiratory syncytial virus (RSV) has been shown to promote the M1 phenotype in AMs during the acute phase of infection; it then stimulates the activation of SDH in the TCA cycle. This, in turn, facilitates the release of cytokines, such as IL-1β, through the HIF-1α/NLRP3 pathway and contributes to the host defense response, stimulating lung tissue inflammation or injury. Conversely, quercetin inhibits SDH activity by promoting itaconic acid anabolism, thus acting to ameliorate lung inflammation (An et al., 2024) (Figure 2).

#### 1.3.3 The influence between the TCA cycle and fungal infection

Fungal infections are relatively uncommon in individuals with intact immune systems. However, *Cryptococcus gattii* has been reported to cause pneumonia in such individuals. Additionally, the decreased expression of proteins associated with the TCA cycle in the lungs of rats infected with *C. gattii* indicates that infections with this fungus may result in the dysregulation of the TCA cycle (Rosa et al., 2019). It has been demonstrated that conditions such as immunodeficiency or the frequent use of antibiotics can promote fungal infections. A study involving mice (Lyz2cre HIF1αFL/FL) revealed that following *Histoplasma capsulatum* infection, those lacking hypoxia-inducible factor 1α (HIF-1α) expression in bone marrow cells began to exhibit increased fungal loads on the third day of infection and ultimately died from the sublethal inoculum of *H. capsulatum* (Fecher et al., 2016). Further mechanistic studies revealed that in the absence of HIF-1α, lung infection by *H. capsulatum* resulted in the accumulation of the TCA-circulating intermediate fumarate, which further promoted IL-10 protein production by inhibiting JmjC domain-containing histone demethylase 5 (KDM5)-mediated histone demethylation. This process downregulates miR-27a expression and further promotes IL-10 protein production, leading to the alleviation of IL-10 transcript degradation. As an anti-inflammatory cytokine, IL-10 accumulation inhibits the ability of macrophages to respond to proinflammatory cytokines which in turn facilitates uninhibited fungal growth and is a primary contributor to elevated host mortality rates (Evans et al., 2021).

In conclusion, the TCA cycle plays a significant role in the progression of infectious lung diseases, their treatment, and posttreatment resistance. However, the mechanism of action of the TCA cycle varies for different pathogens, necessitating further in-depth studies on the basis of the main prevalent pathogens according to local epidemiological statistics.

### 1.4 The TCA cycle and pulmonary vascular disease

**FIGURE 3:**
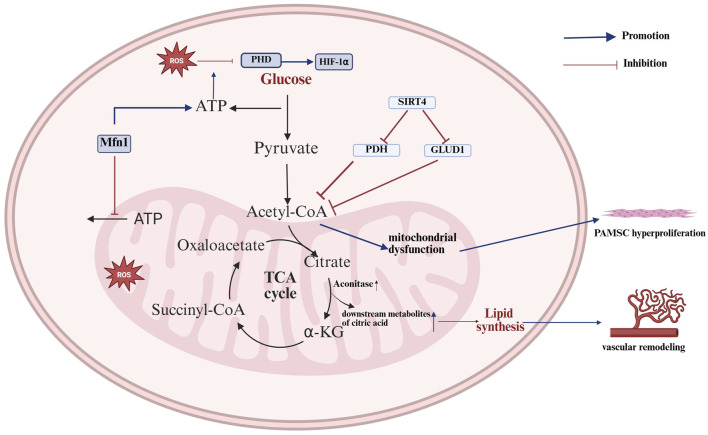
Mechanisms of the TCA cycle in pulmonary vascular disease. The TCA cycle promotes vascular remodeling and smooth muscle hyperproliferation in pulmonary arterial hypertension, primarily by affecting energy production and ROS accumulation, thereby influencing disease progression. Mfn1 results in an excessive degree of mitochondrial fusion, which in turn leads to a decrease in basal ATP and an increase in glycolytic ATP; increased glycolytic ATP production promotes the inhibitory effect of ROS on PHD expression, which further promotes HIF-1α expression and affects PH progression. The increase in aconitase activity results in increased levels of downstream metabolites of citric acid, including isocitrate, succinate, and succinylcarnitine. These metabolites are continuously transported from the mitochondria to the cytoplasm, which in turn increases fatty acid synthesis and thereby promotes vascular remodeling. In chronic hypoxia, the activation of SIRT4 results in the inhibition of the expression of its downstream regulators, PDH and GLUD1. This, in turn, reduces the amount of amino acids entering the TCA cycle, leading to a reduction in TCA cycling activity and mitochondrial dysfunction. Ultimately, this process promotes the over-proliferation of PMSCs (The blue arrows represent promotion, and the red arrows represent inhibition; created using BioRender.com).

Pulmonary vascular diseases encompass a range of conditions, including PH, pulmonary embolism (PE) and pulmonary infarction. PH, in particular, is a pulmonary vascular disease typified by intricate all-vessel pathology, primarily manifested as extensive remodeling and obstruction of the lumen of small arteries (Maron et al., 2021). PH progression involves the aberrant proliferation and dysregulation of multiple cell types, coupled with the development of inflammatory and fibrotic processes throughout the vascular system (Hu et al., 2020). An elevation in pulmonary artery pressure can impose a significant hemodynamic burden on the right ventricle, potentially leading to right ventricular hypertrophy and, in some cases, progression to right heart failure (Provencher et al., 2024). Nevertheless, the etiology and specific pathogenesis of PH remain incompletely understood in the current literature. A metabolomic analysis of hypertrophied right ventricles in male rats from previous studies revealed a trend toward decreased levels of alanine, argininosuccinic acid, and downstream TCA cycle intermediates (including fumaric acid and malic acid), as well as reduced activation of the TCA cycle in rats with PH compared with control rats (Sakao et al., 2021). In contrast, in humans, metabolite analyses of lung samples obtained from patients with PH at the time of lung transplantation *versus* normal lung tissue obtained from cancer patients undergoing surgical lobectomy revealed higher levels of the TCA cycle intermediates citrate and succinate in PH lung tissue than in normal lung tissue (Zhao et al., 2014). In the initial stages of PH, the overexpression of Mitofusin 1 (Mfn1) in pulmonary endothelial cells results in an excessive degree of mitochondrial fusion, which in turn leads to a reduction in the accumulation of TCA cycle intermediates, including succinate, α-KG, and others, leading to a decrease in basal ATP and an increase in glycolytic ATP, resulting in a Warburg effect, which can lead to the activation of mt-ROS-mediated HIF-1α proteins, ultimately influencing the progression of PH (Yegambaram et al., 2024). After progression to severe PH, the activity of aconitase (the enzyme that catalyzes the formation of cis-aconitate from citrate) in the TCA cycle increases, thereby promoting the conversion of citrate to isocitrate and consequently increasing the concentration of metabolic intermediates downstream of citrate. Concurrently, other TCA metabolites, including succinate and succinylcarnitine, are also elevated in PH, thereby promoting the TCA cycle. This results in the continuous translocation of metabolic intermediates of the TCA cycle from the mitochondria to the cytoplasm, which in turn increases fatty acid synthesis and thereby promotes vascular remodeling (Zhao et al., 2014). Nevertheless, in the context of prolonged hypoxia, Sirtuin 4 (SIRT4) is activated in pulmonary artery smooth muscle cells (PSMCs), leading to the inhibition of its downstream regulators, pyruvate dehydrogenase (PDH) and glutamate dehydrogenase 1 (GLUD1). This, in turn, results in a reduction in amino acids, as well as acetyl-CoA and α-KG. Succinyl-CoA, fumarate, and oxaloacetate, which enter the TCA cycle, result in decreased TCA cycle activity and mitochondrial dysfunction, which ultimately leads to the hyperproliferation of PASMCs and the formation and progression of PH. Conversely, exosomes secreted by human-derived mesenchymal stem cells (MSCs) are rich in metabolic proteins and genes and can alleviate PH through the inhibition of SIRT4 and the reestablishment of the TCA cycle and mitochondrial metabolism (Hogan et al., 2019). PE represents the third most prevalent form of cardiovascular disease, following coronary artery disease and stroke (Huang et al., 2025). There are notable disparities in short-term mortality rates across different risk categories for PE (Chowdhury et al., 2023). Metabolomic analysis of serum from patients with different levels of risk for PE revealed significant differences in the levels of the circulating TCA intermediates α-KG, malate, isocitrate, fumarate and cis-aconitate between low-risk and intermediate-high-risk PE patients, with α-KG showing the most significant changes (Zeleznik et al., 2018) (Figure 3).

In addition to the aforementioned studies, recent research on the impact of the TCA cycle on pulmonary vascular diseases has focused primarily on PH. The findings indicate that the TCA cycle has a considerable influence on the progression of PH, suggesting novel insights into the mitigation of PH progression and associated therapeutic approaches. Nevertheless, the number of studies conducted on PE and pulmonary infarction is relatively limited, and further investigations into the progression of these two diseases are warranted.

### 1.5 The TCA cycle and lung cancer

Lung cancer (LC) is the leading cause of cancer-related mortality on a global scale (Siegel et al., 2024). Despite significant advancements in therapeutic interventions, including surgery, chemotherapy, targeted therapies, immunotherapy and radiotherapy, the overall prognosis of patients with LC remains poor. Imbalances in redox homeostasis and disruptions in redox signaling are common features of tumors and play crucial roles in malignant progression and treatment resistance (Mendes et al., 2024). By analyzing microarray datasets and performing metabolomic analyses of plasma from non-small cell lung cancer (NSCLC) patients and healthy individuals, researchers were able to ascertain that NSCLC is primarily associated with the TCA cycle, RNA degradation, and pyrimidine metabolism (Zhao et al., 2020). Patients with NSCLC exhibit elevated plasma levels of oxaloacetate and malate (Cang et al., 2022). Additionally, research has revealed a correlation between mutations in succinate dehydrogenase, fumarate hydratase, and isocitrate and the progression and prognosis of NSCLC (Guo et al., 2015).

#### 1.5.1 The influence of the TCA cycle on the progression of lung cancer

**FIGURE 4:**
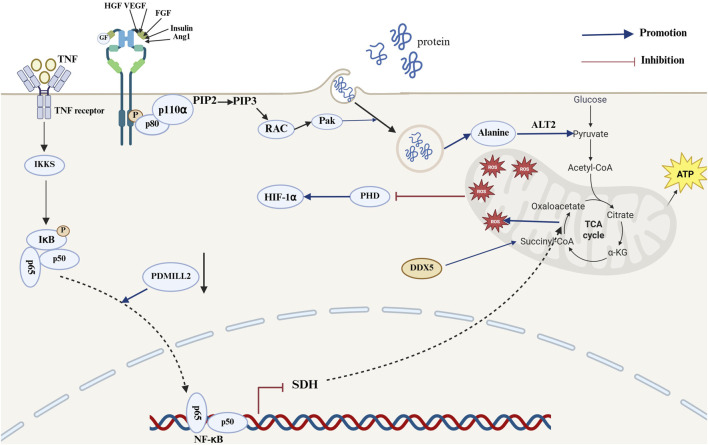
Mechanisms of the TCA cycle in the development of lung cancer. In the mechanism of lung cancer progression, the NF-κB and PI3K signaling pathways can promote lung cancer development by affecting succinate and ROS accumulation and pyruvate production. Activation of the PI3K signaling pathway promotes activation of the Rac-Pak pathway, thereby promoting the megacytosis of extracellular proteins. Proteins transported into the cell are degraded by internalization, resulting in the production of alanine, which can be converted to pyruvate by ALT2 and promote the TCA cycle and gluconeogenesis for energy production. PDMIL2 downregulation promotes impaired SDH expression and increased mt-ROS production following NF-κB pathway activation. The accumulation of mt-ROS results in the inactivation of PHD, which then leads to the activation of HIF-1α. This ultimately promotes the progression of NSCLC. DDX5 promotes succinyl accumulation to promote mitochondrial respiration (The blue arrows represent promotion, and the red arrows represent inhibition; created using BioRender.com).

The rapid proliferation of tumor cells necessitates the production of large quantities of ATP, nucleotides, lipids and proteins, which in turn results in metabolic reprogramming (Paes de Araújo et al., 2019). The rapid uptake of glucose for energy and macromolecules is a crucial aspect of NSCLC progression. The TCA cycle plays a pivotal role in cellular oxidative phosphorylation (Zhang et al., 2024a). Additionally, glycolysis and glucose oxidation via the PDH and TCA cycles are increased in NSCLC relative to neighboring benign lungs (Hensley et al., 2016). Nevertheless, in the absence of glucose, glucose-independent NSCLC cells can activate Rac-Pak signaling via the PI3K signaling pathway and further internalize extracellular proteins through megacellular effluent. Additionally, alanine generated from the degradation of internalized proteins can be converted to pyruvate via alanine transaminase 2 (ALT2), promoting the TCA cycle and gluconeogenesis, which in turn supports NSCLC cell growth and progression (Hodakoski et al., 2019). Furthermore, the downregulation of Postsynaptic density-Discs large-Zonula occludens-1 (PDZ) and Lin11, Isl-1 and Mec-3 (LIM) domain 2 (PDMIL2) expression can result in the impairment of SDH expression and mitochondrial dysfunction within the TCA cycle through the activation of the NF-κB signaling pathway, which in turn leads to the accumulation of succinate and mt-ROS (Yang et al., 2024). The accumulation of mt-ROS and cancer metabolites can additionally facilitate the progression of NSCLC through the inactivation of prolyl hydroxylase (PHD) and the activation of HIF-1α (Dalla Pozza et al., 2020; Yang et al., 2024). Additionally, DEAD-box helicase 5 (DDX5) has been shown to increase mitochondrial respiration by facilitating the accumulation of succinate, an intermediate in the TCA cycle. This, in turn, has been shown to increase the energy requirements of small cell lung cancer, thereby promoting its growth and metastasis (Xing et al., 2020) (Figure 4) The TCA cycle plays a dual role in LC progression, exerting a promoting effect when its activity is increased and a restraining influence when its activity is reduced. Bone morphogenetic protein 2 (BMP2) is markedly expressed in 98% of NSCLC cases. BMP signaling has been shown to increase cell survival, migration, cancer stem cell self-renewal and metastasis, whereas increased BMP expression is associated with poorer survival outcomes (Langenfeld et al., 2003; Huang et al., 2021). BMP signaling can inhibit AMPK signaling by suppressing liver kinase B1 (LKB1); this results in a reduction in TCA cycle intermediates and amino acid expression in the lung adenocarcinoma (LUAD) cell line H1299, which in turn decreases the activity of the TCA cycle and ultimately promotes the progression of NSCLC (Vora et al., 2022). There are numerous mechanisms by which the TCA cycle influences the progression of LC, including those described above; the other potential mechanisms are outlined in Table 1.

**TABLE 1:** Mechanisms by which the TCA cycle influences the progression of LC.

Mechanisms that influence the progression of LC	References
The binding of acetylated signal transducer and activator of transcription 3 (STAT3) to pyruvate decarboxylase facilitates the conversion of pyruvate to acetyl-CoA, thereby enhancing the activity of the TCA cycle and consequently promoting the progression of NSCLC.	Xu et al. (2016)
In NSCLC, low expression of COX7A1 has been shown to reduce the activity of the TCA cycle by inhibiting the expression of α-KG as well as succinate, thereby further attenuating cellular sensitivity to cysteine deprivation-induced iron death	Feng et al. (2022)
A decrease in oxalacetic acid results in a reduction in the abundance and interconversion of TCA cycle intermediates, which subsequently diminish the extent of starvation-induced mitochondrial respiration and cytosolic glutathione consumption in starved NSCLC cells, thus maintaining glutathione redox homeostasis and ultimately influencing NSCLC colony-forming capacity	Bluemel et al. (2021)
NONO regulates the expression of TCA cycle intermediate metabolites by promoting NAMPT expression, which in turn enhances the production of energy metabolism and promotes proliferation in LC.	Kim et al. (2020)
Sustained NRF2 activation inhibits the transcription levels of miR-1 and miR206 in LC cells by promoting the expression of HDAC4, which in turn leads to an increase in glucose flux as well as the production of acetyl-CoA in the TCA cycle, and ultimately promotes LC cell growth	Singh et al. (2013)
The inhibition of NRF2 results in the phosphorylation of focal adhesion kinase and the subsequent reduction in the activity of the mitochondrial, ETC., complex I by negatively regulating the expression of FOCAD, which in turn leads to a decrease in the expression of TCA cycle intermediates α-KG and succinate. Additionally, the activity of the TCA cycle is attenuated, which ultimately reduces the susceptibility of NSCLC cells to cysteine deprivation-induced iron death and promotes NSCLC progression	Liu et al. (2020a)
The exposure of carbon black nanoparticles to A549 cells results in the disturbance of the TCA cycle, characterized by an increase in succinic acid and phosphoenolpyruvic acid and a decrease in cis-aconitic acid and isocitric acid in A549 cells	Hou et al. (2020)

#### 1.5.2 The influence of the TCA cycle on the treatments of lung cancer

Despite the growing number of early diagnostic and therapeutic options for LC, LC remains the leading cause of cancer-related deaths worldwide. Consequently, further research into therapeutic modalities is essential. Immunotherapy represents a significant advancement in the treatment of LC. In patients treated with immunotherapy as a first-line approach, the difference in TCA cycle activity was most pronounced between the long-term and short-term survival groups, with the greatest disparity observed between citrate and cis-aconitate (Xu Y. et al., 2024). Studies have demonstrated that the levels of the TCA cycle intermediates succinate, fumarate, and malate are elevated in LC cells and that their abundance is reduced by the immunotherapeutic agent nivolumab (Alosaimi et al., 2024). Chemotherapy represents a key instrument in the treatment of LC. A metabolomic analysis of the relationship between pemetrexed and LC revealed substantial metabolomic alterations in the TCA cycle pathway in newly diagnosed NSCLC patients compared with healthy controls. Furthermore, significant changes in the pyruvate metabolic pathway have been observed in pemetrexed-treated patients who respond effectively to pemetrexed therapy compared with newly diagnosed NSCLC patients (Sun et al., 2023). Further mechanistic studies revealed that malic acid, a cyclic intermediate of the TCA cycle, can inhibit glucose-6-phosphate (G6PD) activity by binding to malate dehydrogenase 2 (MDH2) and further increase intracellular ROS levels and inhibit DNA synthesis via the pentose phosphate pathway (PPP), which in turn promotes the growth of LC. While dimethyl malate (DMM) can increase the sensitivity of LC cells to chemotherapy, the simultaneous treatment of LC cells with DMM and cisplatin (CDDP) can more significantly inhibit the growth and colony formation of tumor cells, reduce the concentration of chemotherapeutic drugs and decrease the side effects of drugs (Sun et al., 2024). Other mechanisms of the TCA cycle for lung cancer treatment are shown in Table 2.

**TABLE 2:** Mechanisms by which the TCA cycle influences the treatment of LC.

Mechanisms that influence the treatment of LC	References
Mitochondrial division inhibitor-1 treatment inhibits oxidative metabolism and thus lung cancer cell proliferation by mediating pathological ROS production and alterations in isocitrate dehydrogenase activity to reduce circulating levels of TCA.	Dai et al. (2020)
Ym155 binds to mitochondrial DNA, resulting in a reduction in the abundance of TCA cycle intermediates, including α-KG, fumaric acid and malic acid. This appears to be due to the inhibition of, ETC., which in turn affects the activity of the TCA cycle. The consequence of this is an increase in mitochondrial permeability, which ultimately inhibits the growth and promotes the death of NSCLC.	Mondal et al. (2022)
Yeast-derived whole β-glucan particle reduces α-KG production by inhibiting IDH expression through mediating the downregulation of c-MAF expression in macrophages, thereby attenuating TCA cycle activity and polarizing macrophages toward the antitumor M1-type, and ultimately exerting antitumor effects	Liu et al. (2020b)
Docetaxel plays an antitumor role by inhibiting the expression of IDH and lowering the levels of circulating TCA intermediates such as succinic acid, citric acid and α-KG, thereby decreasing the circulating TCA intermediate activity in NSCLC.	Sun et al. (2019)
Fuzi alkaloids play a therapeutic role by converting the metabolic pathway from glycolysis to oxidative phosphorylation in NSCLC by inhibiting PI3K/Akt/mTOR pathway phosphorylation, as well as by promoting the restoration of TCA cycle activity	Zhang et al. (2024b)
BIX reduces the fuel source for the TCA cycle by decreasing the expression level of branched-chain α-keto acid dehydrogenase, which in turn leads to a significant reduction in the level of TCA cycle intermediates as well as the inhibition of mitochondrial metabolism, which ultimately leads to the apoptosis of EGFR-mutated NSCLC cells	Kim et al. (2021)
ATG5 knockdown significantly reduces glucose and lactate uptake in lung tumors, leading to the impaired circulating metabolism and biosynthesis of TCA, as well as increased tumor T cell infiltration, which promotes T cell-mediated tumor killing and comediates antitumor effects	Nunn and Yanxiang Guo (2023)
Citrate inhibits the TCA cycle by inhibiting aconitase, resulting in a significant reduction in TCA cycle metabolites downstream of cis-aconitrate, which in turn exerts an antitumor effect	Ren et al. (2017)

#### 1.5.3 The influence of TCA cycle on the resistance of lung cancer treatments

The high mortality rate of LC is significantly associated with drug resistance. Consequently, delaying drug resistance can markedly improve the prognosis of LC patients. Erlotinib is a targeted drug for epidermal growth factor receptor (EGFR) mutations. However, in comparison with H292 cells, erlotinib-resistant H292 cells demonstrated a marked dependence on glutamine in the production of TCA-related intermediates, particularly α-KG and citrate (Kim et al., 2022). Mechanistic studies have demonstrated that the expression of miR-147b in LUAD cells results in the disruption of the TCA cycle and activation of the pseudohypoxic response; this subsequently activates a tolerance strategy to defend against EGFR inhibition, ultimately leading to an EGFR-tyrosine kinase inhibitor (EGFR-TKI)-tolerant state (Zhang et al., 2019). The TCA cycle has been shown to promote LUAD homologous recombination (HR) activity by mediating SHFM1, which has been shown to result in a poor prognosis in patients with lung adenocarcinoma and a reduction in immunotherapy efficacy (Xu Z. et al., 2024). Furthermore, exogenous glutamine has been shown to generate lipid-based ROS through the TCA cycle, thereby sensitizing cells to erastin-induced iron death (Gao et al., 2019). While arginine succinate synthetase (ASS1) overexpression inhibits the oxidative TCA cycle and disrupts oxidative pathways in NSCLC cells, it also promotes reduced glutamine carboxylation with transamination, which in turn leads to a reduction in mitochondrial lipid ROS production. This ultimately results in resistance to erastin-induced iron death (Hu et al., 2023).

LC has attracted global attention because of its high incidence and mortality rates. The above studies have demonstrated that there has been a notable increase in research activity focused on the role of the TCA cycle in the development and treatment of lung cancer. Intermediate metabolites of the TCA cycle can be metabolized by the human body, having fewer side effects than other chemotherapy and immunotherapy treatments do. Consequently, there is a clear need for further research to explore the role of TCA cycle metabolites in the treatment of LC. One of the reasons for the high incidence and mortality of LC is the difficulty in early diagnosis. There are relatively few studies on the role of the TCA cycle in the early diagnosis of LC; thus, further research in this area is warranted.

## 2 Conclusion

As the TCA cycle plays an important role in energy production and biosynthesis, increasing attention is being given to the TCA cycle and its metabolic intermediates, with a focus on defining their relationship with disease progression, exploring potential mechanisms of action and providing insight into therapeutic options. Lung disease has attracted worldwide attention as a disease of high morbidity, and a thorough understanding of its pathogenesis is beneficial for subsequent treatment and early prevention. Therefore, an increasing number of researchers are focusing on the relationship between the TCA cycle and lung diseases, with LC as the main research category. Studies have shown that the TCA cycle has an important relationship with LC progression, treatment and drug resistance, providing an important basis for improving the prognosis of LC patients. We hope that the mechanism of action and therapeutic aspects identified in this study will be translated into clinical practice in the future. Moreover, relatively few studies have investigated the relationships between the TCA cycle and other lung diseases, and treatment guidelines are relatively limited. Therefore, we hope that in the future, while we continue to research the relationship between the TCA cycle and LC, we will also make breakthroughs in other lung diseases, such as lung infections and chronic airway inflammation, to improve the prognosis and quality of life of patients.
